# Data on the effects of brewery sludge and blended NPS fertilizer rates on yield and yield components of maize (*Zea Mays* L.) in North Mecha District, Northwestern Ethiopia

**DOI:** 10.1016/j.dib.2023.109707

**Published:** 2023-10-20

**Authors:** Fenta Assefa, Zenebe Gebremedhin, Teferi Alem

**Affiliations:** Department of Plant Sciences, College of Agriculture and Environmental Sciences, University of Gondar, Gondar, Ethiopia

**Keywords:** Blended NPS fertilizer, Brewery sludge, Dataset, Grain yield, Maize

## Abstract

Maize is one of the most important cereal crops in Ethiopia. However, its yield is lower than its potential mainly due to low soil fertility problems. Therefore, this field experiment was conducted during 2021 main rainy season with the objective of improving maize production through application of brewery sludge and blended NPS fertilizers in North Mecha district, Northwestern Ethiopia. Factorial combination of three levels of brewery sludge (0, 10 and 20 t ha^−1^) and four levels of blended NPS fertilizer rates (0, 50, 100 and 150 kg ha^−1^) were laid out in RCBD with three replications. The remaining necessary agronomic practices and crop management activities were undertaken uniformly. The data presented under this dataset article includes phenological Parameters (i.e. Days to 50 % emergence, Days to 50 % tasseling, Days to 50 % silking and Days to 90 % physiological maturity), growth parameters (i.e. Plant height, leaf area and leaf area index) and yield and yield component parameters (i.e. Number of ears plant^−1^, Above ground dry biomass yield, Ear length, Number of rows ear^−1^, Number of kernels row^−1^, Number of kernels ear^−1^, Grain yield, Thousand kernels weight, Stover yield and Harvest index). All the collected data were subjected to analysis of variance (ANOVA) and the analysis was carried out using the SAS version 9.0 software computer program's General Linear Model (GLM) procedure [1]. As described in Montgomery [2], the residuals were examined to verify the normal distribution and homogeneous variance model assumptions on the error terms for each response variable. Because the twelve treatment combinations were randomized within each block, the independence assumption is valid. When a treatment effect was significant, multiple means comparison was performed at a 5 % level of significance using the least significant difference (Fisher's LSD) method to generate letter groupings and correlation analysis was performed using the Pearson correlation procedure found in SAS. This dataset article, therefore gives information about the effects of brewery sludge and blended NPS fertilizer rates on maize productivity. Additionally, it provides the economic feasibility of brewery sludge integrated application with blended NPS fertilizer than sole application of blended chemical fertilizers. Hence, this information can allow other researchers to review the supplement data, methods, and make detailed analysis, which possibly giving rise to new lines of inquiry. This can also give rise to new collaborations and boost the reputation of the present research data within the scientific community and to make it available to everyone around the subject matter to use as they wish.

Specifications Table

**Table:** 

Subject	Agriculture
Specific subject area	Agronomy
Type of data	Table and Figures
How the data were acquired	Data of maize related to phenological, growth, yield and yield component parameters of maize were collected by using measurement under field conditions at plant and plot basis based on the nature of the parameter collected.
Data format	Analyzed mean data and raw data
Description of data collection	*Days to 50* % *emergence, Days to 50* % *tasseling, Days to 50* % *silking, Days to 90* % *physiological maturity, Plant height, leaf area, leaf area index, Number of ears plant^−1^, Above ground dry biomass yield, Ear length, Number of rows ear^−1^, Number of kernels row^−1^, Number of kernels ear^−1^, Grain yield, Thousand kernels weight, Stover yield and Harvest index were obtained as described in this article.*
Data source location	*University of Gondar, Central Gondar, Ethiopia, is the owner of the data presented in this article. The experimental sites is located at 11° 20′ N to 11° 29′ N latitude and 37° 04′ E to 37° 10′ E longitude with an altitude of 1960 meter above sea level*[3]*.*
Data accessibility	Repository name: Open Science Framework With article and the raw data is deposited in the Open Science Framework dataset repository available at: https://osf.io/zh5xy/?view_only=f795a84e48244f23a575fd5a028d0348

## 1 Value of the Data


 

• In Ethiopia, maize production and productivity is below its potential even though higher than other major cereal crops produced in the country. This is due to a combination of several production constraints, the most significant of which is nutrient deficiencies that cause declining soil fertility. Hence, to address this problem of soil fertility and enhancing maize productivity, blended fertilizer sources (such as: NPS, NPSZnB, NPSZnBFe and others) were applied in blanket recommendation. But, currently its cost is not affordable by smallholder farmers. Therefore, this dataset gives information on the response of maize growth, yield and yield components to different rates of blended NPS fertilizer.
• Currently, the number of brewery factories in Ethiopia is increasing. Dashen Beer Limited Company is one among those, based in Gondar, Amhara region. It produces a huge amount of sludge every year and too costly to dispose of and pollutes the environment. But, its reuse and application to agricultural land has unrestricted importance as an organic fertilizer and substitute mineral fertilizer to increase crop productivity without any adverse environmental effects. And also it is relatively less cost for application to agricultural land by smallholder's farmers in Ethiopia. Because, it contains 20-30 % more nutrients than commonly used organic fertilizer and reduce 40-50 % application of chemical fertilizers. Thus, this dataset also provides valuable information on the effects of brewery sludge (a byproduct of brewery factory) on maize productivity. As, it supplies both macro and micronutrients and improves soil physical (structure) and biological (microorganisms) activity as to increase its nutrient retention capacity. This information, therefore, can help maize producing farmers to recycle brewery sludge to their agricultural land instead of chemical fertilizer as their cost is now a days increased and not affordable by smallholder farmers.
• However, sole application of chemical (Blended NPS) or organic (brewery sludge) fertilizer has its own limitations. Hence, integrated application of brewery sludge and Blended NPS fertilizer has been emerged as source of alternative that reduces production costs while increasing maize productivity. As a result, agronomists can utilize this dataset article to run trials on different crops or the same crop in different soil types and seasons. This could result in increased crop output at cheap or no cost to farmers, as well as improved research operational efficiency among scholars.

## 2 Objective

• To give information on the effects of brewery sludge and blended NPS fertilizer rates on yield and yield components of maize in North Mecha district, Northwestern Ethiopia
• To show the optimum rates of brewery sludge and blended NPS fertilizer in harnessing maize economic production in North Mecha district, Northwestern Ethiopia
• To give valuable data on the economic feasibility of using brewery sludge and blended NPS fertilizer rates in maize production of North Mecha district, Northwestern Ethiopia

## 3 Data Description

**Fig. 1:**
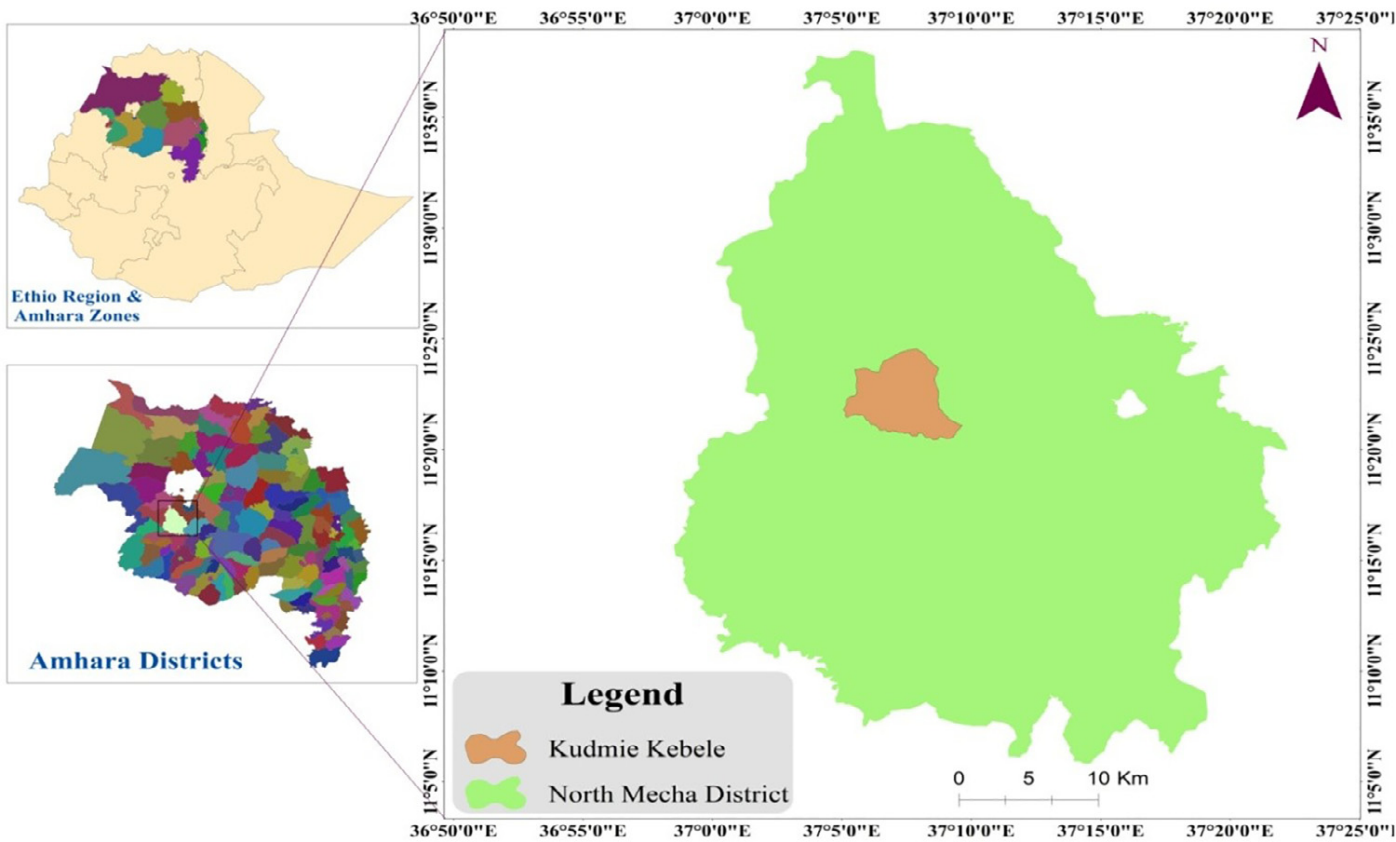
Map of the study area.

**Fig. 2:**
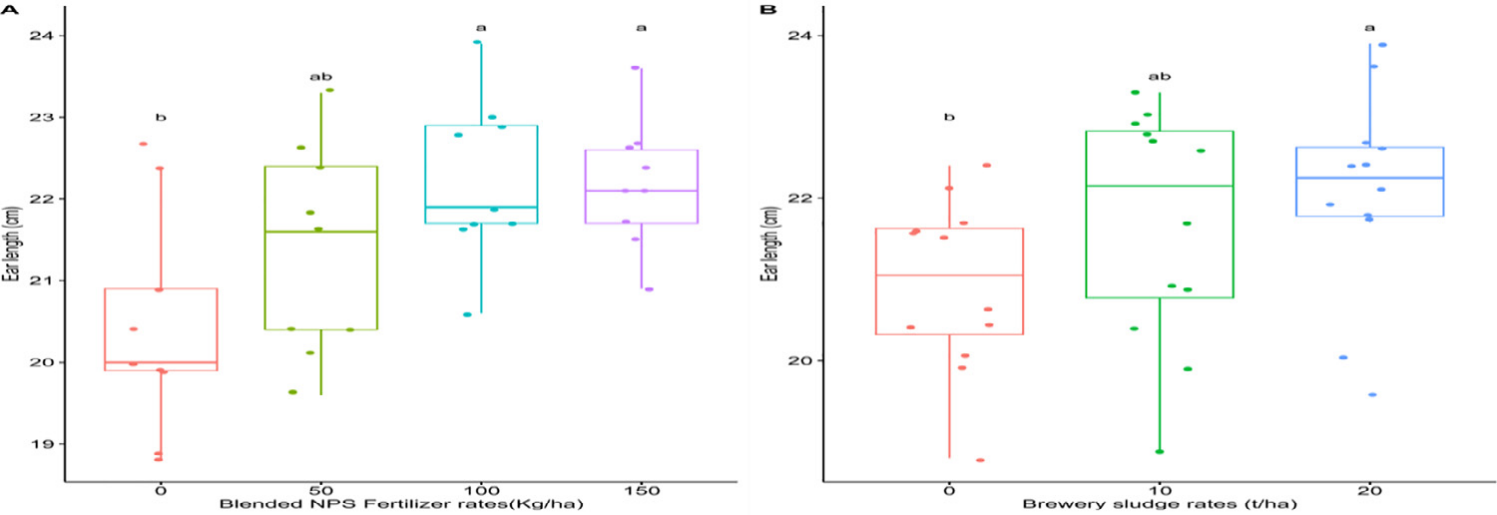
Box plot showing the main effects of blended NPS fertilizer rates and brewery sludge on ear length (cm) of maize.

**Fig. 3:**
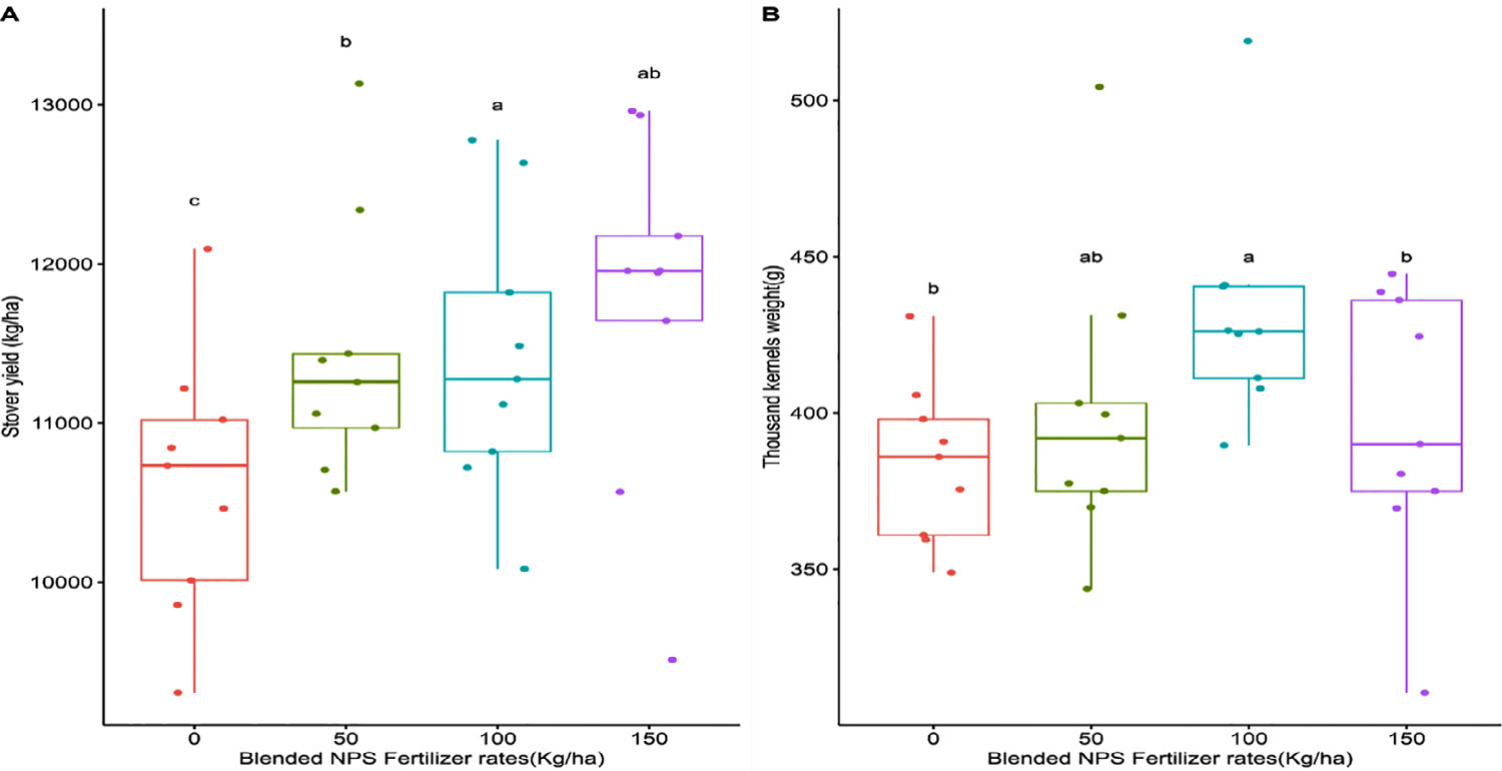
Box plot showing the main effects of brewery sludge and blended NPS fertilizer rates on thousand kernels weight (g) and stover yield (kg ha^−1^) of maize.

**Fig. 4:**
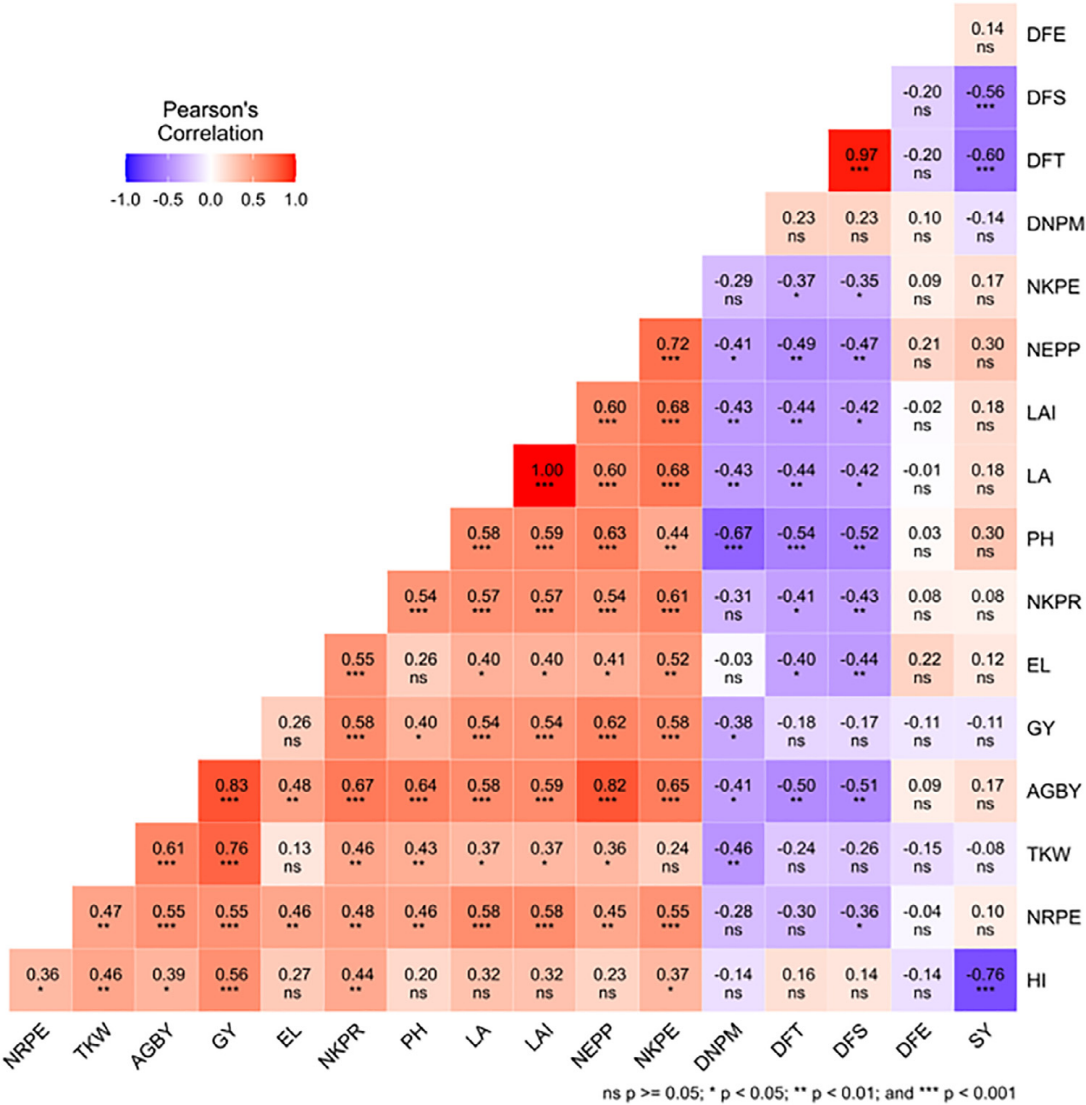
Pearson's correlation matrix graph for agronomic data of maize. **ns** = non- significant at *P* > 0.05; * = significant at *P* ≤ 0.05; ** = highly significant at *P ≤* 0.01; *** = very highly significant at *P ≤* 0.001; **DFE**=days to 50 % emergency; **DFT**=days to 50 % tasseling; **DFS** = days to 50 % silking; **DNPM**=days to 90 % physiological maturity; **PH** = plant height; **LA** = leaf area; **LAI**=leaf area index; **NEPP** = number of ears plant^−1^; **AGDBY**=above ground dry biomass yield; **EL**=ear length; **NRPE**=number of rows ear^−1^; **NKPR**=number of kernels row^−1^; **NKPE**=number of kernels ear^−1^; **GY** = grain yield; **TKW** = thousand kernel weight; **SY** = Stover yield; **HI**=harvest index.

This dataset was collected in a field experiment conducted during the main cropping season from June to December 2021 at Kudmi village of North Mecha district, Northwestern Ethiopia (Fig. 1). The data on boxplot of Fig. 2 shows the main effects blended NPS fertilizer and brewery sludge rates on ear length (cm) of maize. Fig. 3 shows also the main effects of brewery sludge and blended NPS fertilizer rates on thousand kernels weight (g) and stover yield (kg ha^−1^) of maize. Table 1 illustrates the main effects of blended NPS fertilizer and brewery sludge rates on phenological (i.e. Days to 50 % emergence, 50 % tasseling, 50 % silking and 90 % physiological maturity) parameters of maize. The interaction effects of brewery sludge and blended NPS fertilizer rates on growth parameters (i.e. plant height (cm), leaf area (cm^2^), leaf area index) of maize are indicated in Table 2, Table 3, Table 4 respectively. Yield and yield component parameters (i.e. number of ears plant^−1^ in Table 5, above ground dry biomass yield (kg ha^−1^) in Table 6, number of rows ear^1^ in Table 7, number of kernels row^1^ in Table 8, number of kernels ear^1^ in Table 9, grain yield (kg ha-1) in Table 10 and harvest index ( %) in Table 11) of maize are also affected by the interaction of brewery sludge and blended fertilizer rates. The dataset presented in this article shows that, grain yield of maize was strongly and positively correlated with most of the agronomic parameters of maize as shown in Pearson's correlation matrix graph (Fig. 4). The partial budget analyzed dataset for maize production as influenced by brewery sludge and blended NPS fertilizer rates is also presented in Table 12. This dataset article provides the raw data for parameters collected in the field experiment and thus, the raw data is deposited in the OSFHOME dataset library (https://osf.io/42wn5).

**Table 1:** The main effect of brewery sludge and blended NPS fertilizer rates on phenological parameters of maize.

Brewery sludge (t ha^−1^)	DE	DT	DS	DPM
0	7.58	90.91	95.08	162.83**^a^**
10	7.41	89.42	93.58	151.25**^b^**
20	7.92	89.42	93.17	154.50**^b^**
LSD (0.05)	Ns	Ns	Ns	6.46**
NPS Fertilizers (kg ha^−1^)				
0	7.56	93.89**^a^**	98.89**^a^**	157.33
50	7.89	90.22**^b^**	94.33**^b^**	153.33
100	7.89	88.22**^bc^**	91.89**^bc^**	154.33
150	7.78	87.33**^c^**	90.67**^c^**	159.78
LSD (0.05)	Ns	2.66***	3.02***	Ns
CV ( %)	9.34	3.02	3.29	4.89

Means within a column followed by the same letter(s) are not significantly different; *** = very highly significant at *P ≤* 0.001; ** = highly significant at *P ≤* 0.01; * = significant at *P ≤* 0.05; **Ns** = non-significant at *P* > 0.05; **DE**=days to 50 % emergency; **DT**=days to 50 % tasseling; **DS**=days to 50 % silking; **DPM**=days to 90 % physiological maturity**; LSD**= least significant difference**; CV** =Coefficient of variance.

**Table 2:** Interaction effects of brewery sludge and blended NPS fertilizer rates on plant height (cm) of maize.

Brewery sludge rates (t ha^−1^)	Blended NPS Fertilizer rates (Kg ha^−1^)			
	0	50	100	150
0	220.33^c^	242.33^bc^	264.67^ab^	262.33^ab^
10	251.67^b^	266.33^ab^	284.67^a^	256.67^b^
20	267.67^ab^	247.33^b^	255.33^b^	248.33^b^
LSD (0.05)	26.16*			
CV ( %)	6.04			

Means in column and row with the same letter(s) are not significantly different at 5 % level of significance; **LSD** = Least significance difference;* = significant at *P ≤* 0.05; **CV** = Coefficient of variation.

**Table 3:** Interaction effects of brewery sludge and blended NPS fertilizer rates on leaf area (cm^2^) of maize.

Brewery sludge rates (t ha^−1^)	Blended NPS Fertilizer rates (Kg ha^−1^)			
	0	50	100	150
0	3789.80 ^f^	4351.10^e^	5630.00^a^	4767.50^cde^
10	5019.80^bcd^	5343.90^ab^	5650.00^a^	5447.20^ab^
20	5306.50^abc^	5262.30^abc^	4928.80^bcd^	4643.20^de^
LSD (0.05)	541.17***			
CV ( %)	6.38			

Means in column and row with the same letter(s) are not significantly different at 5 % level of significance; **LSD** = Least significance difference; *** = very highly significant at *P ≤* 0.001; **CV** = Coefficient of variation.

**Table 4:** Interaction effects of brewery sludge and blended NPS fertilizer rates on leaf area index of maize.

Brewery sludge rates (t ha^−1^)	Blended NPS Fertilizer rates (Kg ha^−1^)			
	0	50	100	150
0	1.68^f^	1.94^e^	2.50^a^	2.12^cde^
10	2.23^bcd^	2.37^ab^	2.51^a^	2.42^ab^
20	2.36^abc^	2.34^abc^	2.19^bcd^	2.07^de^
LSD	0.24***			
CV ( %)	6.35			

Means in column and row with the same letter(s) are not significantly different at 5 % level of significance; **LSD** = Least significance difference; *** = very highly significant at *P ≤* 0.001; CV ( %) = Coefficient of variation.

**Table 5:** Interaction effects of brewery sludge and blended NPS fertilizer rates on number of ears per plant of maize.

Brewery sludge rates (t ha^−1^)	Blended NPS Fertilizer rates (Kg ha^−1^)			
	0	50	100	150
0	1.13^d^	1.43^cd^	1.73^bc^	1.67^bc^
10	1.50^bc^	1.77^bc^	2.37^a^	1.60^bc^
20	1.67^bc^	1.80^b^	1.83^b^	1.60^bc^
LSD (0.05)	0.34*			
CV ( %)	12.08			

Means in column and row with the same letter(s) are not significantly different at 5 % level of significance; **LSD** = Least significance difference; * = significant at *P ≤* 0.05; CV ( %) = Coefficient of variation.

**Table 6:** Interaction effects of brewery sludge and blended NPS fertilizer rates on above ground dry biomass yield (kg ha^−1^) of maize.

Brewery sludge rates (t ha^−1^)	Blended NPS Fertilizer rates (Kg ha^−1^)			
	0	50	100	150
0	15582.50^g^	17628.00^ef^	19395.90^bc^	18931.80^cbd^
10	17268.80^f^	18475.10^bcde^	21704.80^a^	18320.80^cdef^
20	17893.60^def^	19574.40^b^	18771.10^bcde^	18235.90^cdef^
LSD (0.05)	1174.60***			
CV ( %)	3.75			

Means in column and row with the same letter(s) are not significantly different at 5 % level of significance; **LSD** = Least significance difference; *** = very highly significant at *P ≤* 0.001; **CV** ( %) = Coefficient of variation.

**Table 7:** Interaction effects of brewery sludge and blended NPS fertilizer rates on number of rows per ear of maize.

Brewery sludge rates (t ha^−1^)	Blended NPS Fertilizer rates (Kg ha^−1^)			
	0	50	100	150
0	12.40^c^	12.40^c^	14.00^ab^	13.73^ab^
10	12.53^c^	13.20^bc^	14.27^a^	13.20^bc^
20	13.87^ab^	14.00^ab^	13.60^ab^	13.67^ab^
LSD (0.05)	1.04*			
CV ( %)	4.56			

Means in column and row with the same letter(s) are not significantly different at 5 % level of significance; **LSD** = Least significance difference; *= significant at *P ≤* 0.05; **CV** = Coefficient of variation.

**Table 8:** Interaction effects of brewery sludge and blended NPS fertilizer rates on number of kernels per row of maize.

Brewery sludge rates (t ha^−1^)	Blended NPS Fertilizer rates (Kg ha^−1^)			
	0	50	100	150
0	37.93^d^	42.67^bc^	44.47^ab^	44.07^abc^
10	41.27^c^	41.80^bc^	46.27^a^	42.67^bc^
20	44.73^ab^	44.13^abc^	43.93^abc^	43.47^abc^
LSD (0.05)	3.03*			
CV ( %)	4.15			

Means in column and row with the same letter(s) are not significantly different at 5 % level of significance; **LSD** = Least significance difference; *= significant at *P ≤* 0.05; **CV** ( %) = Coefficient of variation.

**Table 9:** Interaction effects of brewery sludge and blended NPS fertilizer rates on number of kernels per ear of maize.

Brewery sludge rates (t ha^−1^)	Blended NPS Fertilizer rates (Kg ha^−1^)			
	0	50	100	150
0	478.40^d^	517.73^cd^	582.33^ab^	566.07^abc^
10	538.00^bc^	563.33^abc^	616.80^a^	533.13^bcd^
20	595.20^a^	582.47^ab^	583.80^ab^	535.07^bcd^
LSD (0.05)	56.93*			
CV ( %)	6.03			

Means in column and row with the same letter(s) are not significantly different at 5 %  level of significance; **LSD** = Least significance difference; * = significant at *P ≤* 0.05; **CV** = Coefficient of variation.

**Table 10:** Interaction effects of brewery sludge and blended NPS fertilizer rates on grain yield (kg ha^−1^) of maize.

Brewery sludge rates (t ha^−1^)	Blended NPS Fertilizer rates (Kg ha^−1^)			
	0	50	100	150
0	6232.0^g^	6441.5^fg^	7831.2^bc^	7339.4^cde^
10	6601.1^efg^	7680.4^bcd^	9163.4^a^	6840.0^defg^
20	7209.1^cdef^	8265.5^b^	7078.9^cdefg^	6818.3^defg^
LSD (0.05)	866.9***			
CV ( %)	7.02			

Means in column and row with the same letter(s) are not significantly different at 5 % level of significance; **LSD** = Least significance difference; *** = very highly significant at *P ≤* 0.001; CV (%) = Coefficient of variation.

**Table 11:** Interaction effects of brewery sludge and blended NPS fertilizer rates on harvest index ( %) of maize.

Brewery sludge rates (t ha^−1^)	Blended NPS Fertilizer rates (Kg ha^−1^)			
	0	50	100	150
0	39.98^abcd^	36.57^d^	40.37^abc^	38.78^abcd^
10	38.23^bcd^	41.53^ab^	42.17^a^	37.34^cd^
20	40.25^abcd^	42.23^a^	37.70^cd^	37.38^cd^
LSD (0.05)	3.68*			
CV ( %)	5.53			

Means in column and row with the same letter(s) are not significantly different at 5 % level of significance; **LSD** = Least significance difference; *= significant at *P ≤* 0.05; CV (%) = Coefficient of variation.

**Table 12:** Partial budget analysis for maize yield as influenced by brewery sludge and blended NPS fertilizer rates for maize crop production.

BS (t ha^−1^)	NPS (Kg ha^−1^)	UAGY (kg ha^−1^)	AGY (kg ha^−1^)	TGB (ETB ha^−1^)	TVC (ETB ha^−1^)	NB (ETB ha^−1^)	MRR (%)
0	0	6232.0	5608.80	100958.4	0	100958.4	–
10	0	6601.1	5940.99	106937.8	703	106234.8	750.55
0	50	6441.5	5797.35	104352.3	1077	103275.3	D
20	0	7209.1	6488.19	116787.4	1406	115381.4	1301.08
10	50	7013.7	6912.33	124422.5	1780	122642.5	1941.47
0	100	8164.6	7348.14	126865.4	2154	124711.4	553.18
20	50	8265.5	7438.95	133901.1	2483	131418.1	2038.51
10	100	9163.4	8247.06	148447.1	2857	145590.1	3789.30
0	150	7339.4	6605.46	118898.3	3231	115667.3	D
20	100	7078.9	6371.01	114678.2	3560	111118.2	D
10	150	6840.0	6156.00	110808.0	3934	106874	D
20	150	6818.3	6136.47	110456.5	4637	105819.5	D

**BS**= brewery sludge; **ETB**= Ethiopian Birr; **UAGY**= unadjusted Grain Yield; **AGY**=Adjusted Grain Yield; **TGB**= Total Gross benefit; **TVC** = Total variable cost; **NB** = Net benefit; local price of maize=18 ETB kg^−1^; costs of blended NPS fertilizer=19.04 ETB kg^−1^; transportation cost of NPS=130 and its labor cost for application=120 ETB/8hrs/100 kg blended NPS fertilizer; costs of brewery sludge=60 ETB t^−1^; transportation of brewery sludge=60 ETB and application cost=43 ETB/2 hrs and 45 minutes for 10 ton brewery sludge.

## 4 Experimental Design, Materials and Methods

Factorial combinations of three levels of brewery sludge (0, 10 and 20 t ha^−1^) and four levels of blended NPS fertilizer rate (0, 50, 100 and 150 kg ha^−1^) were laid out in RCBD^1^ with three replications. The land was prepared by oxen/animal drawn plowing and hand tools, after that leveled and smoothed by human labor using hand tools. As per the design and treatments, the experimental field was then manually subdivided into blocks and plots based on the design and treatments. An experiment had a total area of 13.25m width× 41.5m length (549.875m^2^) and a gross plot size of 3.75m width x 3m length (11.25m^2^). A 1.0 m wide open path separated the adjacent blocks and a 0.5m wide path separated plots within a block from one another. Using a random table number, experimental treatments were assigned to experimental plots in each block. As a result, there were 5 rows totaling 3m in length, with 10 plants per row and 50 plants per plot. To avoid possible border effects, the net plot size (harvestable area) was 2.25m x 2.4m (5.4m^2^) (i.e. the middle 3 rows from each plot) by excluding 1 outermost row on both sides of each plot horizontally and 0.3m row segment from both ends of the plot vertically. Then, all the remaining necessary agronomic practices and crop management activities (such as, plot preparation, sowing, weeding, harvesting and threshing) were undertaken uniformly.

### 4.1 Crop Data Collected

#### 4.1.1 Phenological parameters

Days to 50 % emergence were recorded from the time of sowing to the date when 50 % of the plants appeared above the ground from each plot. Days to 50 % tasseling were recorded as the number of days from sowing to when 50 % of the plants in each net plot produced tassels. Days to 50 % silking were recorded as the number of days from sowing to when 50 % of the plants in each net plot produced silk. Days to 90 % physiological maturity were recorded as the number of days from sowing to when 90 % of the plants in each net plot formed a black layer at the point where the kernel is attached to the ear.

#### 4.1.2 Growth parameters

Plant height of maize was measured in centimeters as the distance from ground level to the point where the tassel starts to branch and five plants sampled randomly from the net plot used for this purpose at physiological maturity. The mean of five plants were recorded as plant height. Leaf area was measured from all available leaves of five plants per net plot at 50 % silking stage and leaf length and maximum width were measured and calculated by using the following formula;

(1) LA=L×W×K;

Where, LA: Leaf area per plant (cm^2^); L: Length of leaf (cm); W: Width of leaf (cm); K: correction factor (0.75) [4]. Leaf area index (LAI) was calculated as the ratio of the total leaf area of a plant (cm^2^) to the area of land occupied by the plant [5] and was determined using three leaves per plant *i.e.* ear leaves, one above and one below the ear leaf as described by [4], using the following formula;

(2) $$\text{LAI}=\frac{\text{Area}\;\text{of}\;\text{green}\;\text{leaf}\;\text{per}\;\text{plant}}{\text{Area}\;\text{occupied}\;\text{by}\;\text{the}\;\text{plant}}$$

#### 4.1.3 Yield and yield component parameters

Number of ears per plant was counted from five randomly selected plants and the mean was used for the analysis. Above ground dry biomass yield: plants used for grain yield determination were taken after sun drying five plants from the net plot area and weighed to obtain the total biomass yield and expressed in kg ha^−1^. Ear length was measured from five randomly taken plants after harvest from the net plot. Number of rows per ear was counted on five randomly selected ears and the mean values were recorded as number of rows per ear. Number of kernels per row was determined by counting the number of kernels in each grain row of five randomly taken ears from the net plot area at crop harvest and expressed as the mean number per row. Number of kernels per ear were counted from five ears in the net area and the mean was used for analysis. Grain yield was determined from the net harvestable area; the yield was adjusted to 12.5 % moisture content, and the result converted to kg ha^−1^.

(3) $$\text{Grain}\;\text{yield}\;\left(\text{kg}\;ha^{-1}\right)\;\text{at}\;12.5\;\%\;\text{moisture}\;\text{base}=\text{Obtained}\;\text{yield}\;\left(\text{kg}\;ha^{-1}\right)\times \frac{100-\;\%\text{MC}}{100-12.5};$$

Where, MC = grain moisture content. Thousand kernel weights were determined based on the weight of thousand kernels sampled from the sample used to determine the grain yield of each treatment. Counted manually by hand, Electronic balance was used for weighing after adjusted to 12.5 % moisture level. Stover yield was measured by taking the weight of the straw harvested from the net plot area of each plot and converted to kilograms per hectare after sun drying the straw. Harvest index (HI): It was calculated as the ratio of grain yield to total above ground dry biomass yield multiplied by 100 at harvest from the respective treatments [6].

(4) $$\text{HI}\left(\;\%\right)=\frac{\text{Grain}\;\text{yield}}{\text{Aboveground}\;\text{dry}\;\text{biomass}\;\text{yield}\;}\times 100$$

### 4.2 Statistical Data Analysis

The collected data were subjected to analysis of variance (ANOVA) and the analysis was carried out using the SAS version 9.0 software computer program's General Linear Model (GLM) procedure [1]. As described in Montgomery [2], the residuals were examined to verify the normal distribution and homogeneous variance model assumptions on the error terms for each response variable. Because the twelve treatment combinations were randomized within each block, the independence assumption is valid. When a treatment effect was significant, multiple means comparison was performed at a 5 % level of significance using the least significant difference (Fisher's LSD) method to generate letter groupings and correlation analysis was performed using the Pearson correlation procedure found in SAS.

### 4.3 Partial Budget Analysis

A partial budget analysis was performed to investigate the economic feasibility of the treatments (brewery sludge and blended NPS fertilizer). A partial budget, dominance and marginal analysis were used. Partial budget and marginal analysis of economic concepts were used to analyze the economic data. Partial budget is a method of organizing experimental data and information about the costs and benefits of various alternative treatments. Marginal analysis is a method for comparing the costs that vary with the net benefits for selecting the best technology for recommendations from the experiment. The market cost of maize was taken from local market prices in Ethiopian Birr (ETB) kg^−1^ during harvest. Prices of blended NPS fertilizer and brewery sludge and their transportation and application cost were taken as fixed price during sowing.

Partial budget averaged of the twelve (12) treatments calculated from income and expenses based on variable cost (Table 12). The mean yield was adjusted downward by 10 % and was used to reflect the difference between the experimental field and the expected yield from farmers’ fields with farmers’ practices from the same treatments [7]. The gross field benefit was calculated by multiplying the adjusted grain yield of each treatment with the farm price of the crop during harvesting. All variable costs were calculated excluding the price of other agronomic practices such as cost of seed, land plowing, sowing, weeding, protection of the farm and harvesting because it was uniform for all treatments. The variable costs were summed up and subtracted from gross field benefits which were taken as net benefit. Based on the results of the dominance analysis, treatments were chosen in increasing order of total variable costs. For each pair of ranked treatments, the percent marginal rate of return (MRR) was calculated. The MRR ( %) between any pair of un-dominated treatments was the return per unit of investment in fertilizer. It was calculated by dividing the change in net benefit to the change in variable costs. Analysis of marginal rate of return (MRR) was carried out for non-dominated treatments and the MRRs were compared to a minimum acceptable rate of return (MARR) of 100 % to select the optimum treatment [8].

## Ethical Statements

The dataset collected in this study did not involve animals and humans.

## CRediT authorship contribution statement

**Fenta Assefa:** Conceptualization, Methodology, Software, Writing – original draft, Investigation, Formal analysis. **Zenebe Gebremedhin:** Supervision, Writing – review & editing. **Teferi Alem:** Supervision, Writing – review & editing.

## Data Availability

Data on the Effects of Brewery Sludge and Blended NPS Fertilizer Rates on Yield and Yield Components of Maize (Original data) (Open Science Framework)

## Footnotes

1 RCBD= a Randomized Complete Block Design.
